# Comparing four different methods for the management of ectopic pregnancy: A cross-sectional study

**DOI:** 10.18502/ijrm.v20i3.10709

**Published:** 2022-04-21

**Authors:** Zahra Shiravani, Sana Atbaei, Bahia Namavar Jahromi, Mojgan Hajisafari Tafti, Shaghayegh Moradi Alamdarloo, Tahereh Poordast, Adel Noori, Sedighe Forouhari, Soudabeh Sabetian

**Affiliations:** ^1^Gynecology Oncology Division, Department of Obstetrics and Gynecology, School of Medicine, Shiraz University of Medical Science, Shiraz, Iran.; ^2^Maternal-Fetal Medicine Research Center, Shiraz University of Medical Sciences, Shiraz, Iran.; ^3^Department of Obstetrics and Gynecology, School of Medicine, Shiraz University of Medical Sciences, Shiraz, Iran.; ^4^Infertility Research Center, Shiraz University of Medical Sciences, Shiraz, Iran.; ^5^Department of Obstetrics and Gynecology, School of Medicine, Shahid Sadoughi University of Medical Sciences, Yazd, Iran.; ^6^Shiraz University of Medical Sciences, Shiraz, Iran.

**Keywords:** *Ectopic pregnancy*, * Methotrexate, β -hCG*, *Treatment.*

## Abstract

**Background:**

Ectopic pregnancy (EP) is one of the major causes of maternal mortality during the first trimester of pregnancy.

**Objective:**

Four treatment methods for EP including single-dose methotrexate (SD-MTX), double-dose methotrexate, expectant and surgical management were considered.

**Materials and Methods:**

In this cross-sectional study, the clinical characteristics of 365 women aged 15-44 yr who had been diagnosed with EP were reviewed from March 2017 to March 2019 in hospitals affiliated to Shiraz University of Medical Sciences, Shiraz, Iran. Receiver operating characteristics curves were plotted to determine the cut-off points for size of ectopic mass and beta-human chorionic gonadotropin (β-hCG) that suitably discriminated between double-dose methotrexate and surgery management.

**Results:**

The most common site of EP was adnexa. According to the receiver operating characteristics analysis, surgery was the best plan for the women with an ectopic mass > 34.50 mm in diameter or with an initial β-hCG level > 6419 mIU/ml. The β-hCG levels in the women successfully treated with SD-MTX were significantly lower than in those with failed treatment (p = 0.02). The SD-MTX group had a higher success rate and significantly shorter duration of hospitalization, and so this was a more effective medical treatment in comparison with the double-dose protocol.

**Conclusion:**

Surgery is proposed as the best option for the cases with large ectopic mass or high β-hCG level. SD-MTX had a higher success rate and shorter hospital stay than the double-dose protocol, and so was found to be an efficient and safe alternative. Further randomized clinical trials with larger sample sizes are recommended to validate the current results.

## 1. Introduction

Ectopic pregnancy (EP) is defined as implantation of a blastocyst outside the uterine cavity (1). It is a major reason for maternal morbidity and mortality during the first trimester (2). The incidence is about 2% of all pregnancies (3). Although the etiology of EP is not identified clearly, several risk factors such as tubal infection, previous tubal surgery and previous EP increase the probability of this occurrence (4). Usually, an EP is located in one of the fallopian tubes and requires emergent treatment; as it gets large, it can cause tubal rupture due to heavy internal bleeding and hypovolemic shock (5). Today, the options for the treatment of EP include surgery, medical treatment and expectant management (6). Surgery treatment includes salpingectomy, salpingotomy and salpingostomy, which can be performed by laparotomy or laparoscopy (7). Laparoscopy has significant advantages such as shorter hospital stays, quicker recovery and lower cost than laparotomy. Sometimes laparotomy is preferred because of easier access to tissue in hemodynamically unstable cases (8).

In women who are hemodynamically stable, without any sign of rupture of EP, systemic administration of intramuscular methotrexate (MTX) is acceptable by single-dose (SD), double-dose (DD) or multiple dose protocols (9). MTX is contraindicated with renal, hepatic, gastrointestinal or haemopoietic disorders (10). Weekly beta-human chorionic gonadotropin (β-hCG) monitoring follow-up is recommended until levels decline below 15 mIU/ml. If medical treatment is deemed unsuccessful, the patients will need surgical treatment (11).

It is known that some cases of tubal EP with no significant symptoms resolve spontaneously. Therefore, in selected women who are clinically stable with an undiagnosed EP location in ultrasound and a β-hCG less than 1000 mIU/ml, expectant management is a valuable option of treatment.

As there is no consensus on the best management for EP, we aimed to compare the different protocols for treatment of EP in terms of the gestational age, size of ectopic mass, duration of hospital stay, and history of pelvic surgery, pelvic infection, infertility, abortion and EP, as well as site of EP and β-hCG level. In order to discriminate properly between double-dose methotrexate (DD-MTX) and surgery management, we plotted receiver operating characteristics (ROC) curves to determine cut-off points for β-hCG levels and size of ectopic mass.

## 2. Materials and Methods 

### Study design

In this cross-sectional study, the data of 365 women aged between 15-44 yr with the diagnosis of EP were reviewed between March 2017 and March 2019 in hospitals affiliated to Shiraz University of Medical Sciences, Shiraz, Iran.

### Inclusion and exclusion criteria

Women aged 15-44 yr who were diagnosed with EP were included. EP was diagnosed if the serum β-hCG level had a slower doubling time on serial measurement and the gestational sac was not seen in the uterine cavity by transvaginal sonography. The subjects who refused to complete their treatment in the current center or had incomplete historical data in the hospital records were excluded.

### Data collection

Data on participants' demographic, medical and clinical characteristics (age, gestational age, size and location of ectopic mass, duration of hospital stay, serial β-hCG level, type of surgery, and history of EP, infertility, pelvic infection, and pelvic surgery were retrieved from hospital records. The cases were classified according to treatment methods and based on the success of the treatment protocols (response vs. failure).

Surgery was the first plan for the cases with: 1) severe free fluid in the abdominal pelvic cavity and who were unstable hemodynamically; 2) severe abdominal pain; 3) based on participant's preference; or 4) contraindication for medical treatment. The remaining cases were treated by SD- or DD-MTX (50 mg/m^2^, injected intramuscularly; Ebewe company, Austria) on day one, or days one and four, respectively. If the β-hCG level declined ≥ 15% between days four and seven, the MTX therapy was considered to be successful; otherwise, the management was assumed to have failed. Some cases with the criteria of clinical stability were treated by expectant management.

### Ethical considerations

The local Medical Ethics Committee of Shiraz University of Medical Sciences, Shiraz, Iran approved the study protocol (Code: IR.SUMS.REC.1395.S940). All of the enrolled participants signed a written informed consent form after complete explanation of the study design.

### Statistical analysis

The Statistical Package for the Social Sciences (SPSS) version 22 was used to analyze the data. One-way analysis of variance (ANOVA) was used to statistically compare the means of the four treatment groups. Tukey's test was used for a pairwise comparison of all means. The difference between two groups was analyzed by independent sample *t* test. Chi-square test was performed to determine whether there were any statistically significant differences between the descriptive data. Quantitative data were expressed as mean ± SD and the qualitative data were represented as frequencies or percentages. A p-value < 0.05 was considered as significant. ROC curves were generated to assess the optimal cut-off value for the size of ectopic mass and the initial β-hCG that discriminated between DD-MTX and surgery management. The cut-off points were denoted by the value that had the largest sum of sensitivity and specificity.

## 3. Results

Among the 365 women with EP, 87 cases were treated by SD-MTX, 43 by DD-MTX, 154 by surgery, and 81 by expectant management.

According to our results, the most common site of EP was right and left adnexa and there were no significant differences between different treatments. The women who underwent surgery had the largest ectopic mass, the highest β-hCG level, and the shortest hospital stay (p < 0.01) (Table I).

Surgery had the highest success rate (99.35%) and DD-MTX had the lowest (72.09%) (Table I). Among the surgery group, the treatment failed for only one case who had laparoscopy-salpingostomy.

**Figure 1:**
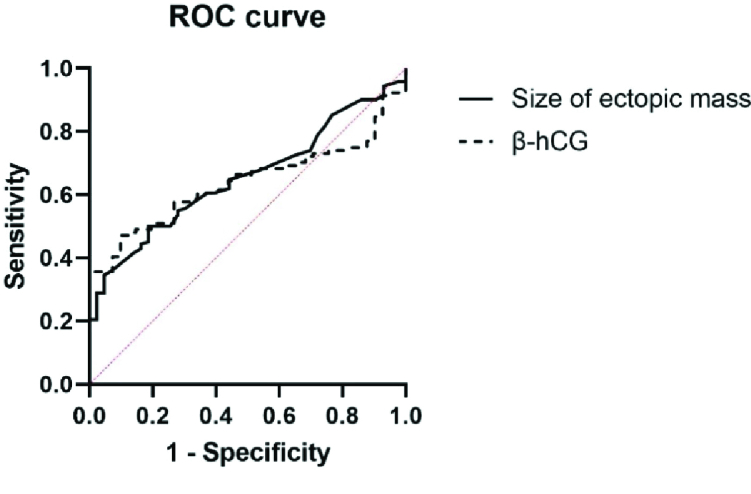
ROC curves for size of ectopic mass and β-hCG level as discriminators between surgery and DD-MTX. Size of ectopic mass AUC: 0.65 (95% CI: 0.57-0.73), P-value < 0.01, β-hCG level AUC: 0.64 (95% CI: 0.55-0.72), P-value < 0.01. AUC: Area under curve, CI: Confidence interval, ROC: Receiver operating characteristics, β-hCG: Beta-human chorionic gonadotropin.

The ROC analysis is presented in figure 1. The areas under the curve for the size of ectopic mass and β-hCG level were statistically significant (p < 0.01). The ROC curves suggested that a size of ectopic mass > 34.50 mm with 42% sensitivity and 67% specificity, and a β-hCG level > 6419 mIU/ml with 47% sensitivity and 90% specificity were suitable cut-off points for selecting surgery as the treatment plan.

The results of comparison between the SD- and DD-MTX groups are summarized in table II. There were no significant differences in the size of ectopic mass, β-hCG level, duration to β-hCG < 5 mIU/ml or treatment success between the two groups. Hospital stays in the SD-MTX group were significantly shorter than in the DD-MTX group (p = 0.02) (Table II).

Within each group, the size of ectopic mass and β-hCG level of the women whose treatment was successful vs. in those whose treatment failed were compared (Table III). Only SD-MTX group represented a significant difference in β-hCG level between successful and failed treatment (p = 0.02).

**Table 1:** Clinical characteristics according to treatment methods


**Groups**		**Single-dose (n = 87)**	**Double-dose (n = 43)**	**Surgery (n = 154)**	**Expectant management (n = 81)**	**P-value**
**Age^a^ (yr)**		29.38 ± 5.89	31.34 ± 5.06	29.98 ± 5.29	30.72 ± 4.44	0.16*
**History^b^**						
	**Pelvic surgery**	35 (40.2)	26 (60.5)	85 (55.2)	47 (58.0)	0.06**
	**Pelvic infection**	2 (2.3)	1 (2.3)	0 (0)	0 (0)	0.14**
	**Infertility**	16 (18.4)	8 (18.6)	30 (19.5)	14 (17.3)	0.98**
	**Abortion**	26 (29.9)	20 (46.5)	63 (40.9)	35 (43.2)	0.18**
	**Ectopic pregnancy**	9 (10.3)	4 (9.3)	14 (9.1)	11 (13.6)	0.75**
**Gestational age^a^ (days)**		50.21 ± 14.63	49.36 ± 14.59	49.90 ± 14.65	46.57 ± 15.89	0.46*
**EP site^b^**						
	**Adnexa**	70 (80.5)	33 (76.7)	127 (82.5)	67 (82.7)	0.83**
	**Cervix**	1 (1.1)	0 (0)	1 (0.6)	0 (0)	0.73**
	**Pelvic cavity**	1 (1.1)	0 (0)	6 (3.9)	0 (0)	0.12**
	**Section site**	2 (2.3)	1 (2.3)	1 (0.6)	0 (0)	0.40**
	**Cornea**	1 (1.1)	1 (2.3)	1 (0.6)	1 (1.2)	0.82**
	**Information was not** **recorded**	12 (13.8)	8 (18.6)	18 (11.7)	13 (16.0)	—
**Size of ectopic mass^a^ (mm)**		27.64 ± 13.13 ${}^{▲}$	27.91 ± 9.04 ${}^{■}$	38.87 ± 20.63	27.74 ± 11.46 ${}^{●}$	< 0.01* ${}^{▲}$ < 0.001*** ${}^{■}$ < 0.001*** ${}^{●}$ < 0.001***
**Initial β-hCG level^a^ (mIU/ml)**		2498.19 ± 5042.40 (2196.0♦)	3300.02 ± 2610.46 (3222.5♦)	12162.30 ± 16628.82 (14476.5♦)	2306.61 ± 4775.57 (1948.0♦)	< 0.01*
**Length time until β-hCG < 5** **mIU/mla (days)^a^**		33.68 ± 14.41	35.88 ± 19.69	NA	NA	0.94*
**Hospital stay^a^ (days)**		8.30 ± 4.00	10.48 ± 5.47	4.36 ± 1.77	5.22 ± 2.88	< 0.01*
**Treatment success^b^**		70 (80.46)	31 (72.09)	153 (99.35)	71 (87.65)	< 0.01**
^a^Data shown as Mean ± SD. ^b^Data shown as n (%). *ANOVA test, **Chi-square test, ***Tukey's test, P < 0.05 as significant, N: Number, EP: Ectopic pregnancy, β-hCG: Beta-human chorionic gonadotropin, ${}^{▲}$ Comparison between single-dose and surgery, ${}^{■}$ Comparison between double-dose and surgery, ${}^{●}$ Comparison between expectant and surgery, ♦IQR: Interquartile range, NA: Not applicable						

**Table 2:** Comparison between single-dose and double-dose


**Variables**	**Single-dose (n = 87)**	**Double-dose (n = 43)**	**P-value**
**Size of ectopic mass^a^ (mm)**	27.64 ± 13.13	27.91 ± 9.04	0.89*
** β-hCG level before treatment^a^ (mIU/ml)**	2498.19 ± 5042.40 (2255.55♦)	3300.02 ± 2610.46	0.34*
**Length time until β-hCG < 5 mIU/mla (days)^a^**	33.68 ± 14.41	35.88 ± 19.69	0.74 *
**Hospital stay^a^ (days)**	8.30 ± 4.00	10.48 ± 5.47	0.02*
**Treatment success^b^**	70 (80.46)	31 (72.09)	0.39**
^a^Data shown as Mean ± SD. ^b^Data shown as n (%). β-hCG: Beta-human chorionic gonadotropin, *Independent sample *t* test, **Chi-square test, βIQR: Interquartile range			

**Table 3:** Size of EP mass and β-hCG level according to treatment success


**Groups**	**Size of EP mass (mm)**		** β-hCG level (mIU/ml)**			
	**Treatment success**	**Treatment failure**	**P-value**	**Treatment success**	**Treatment failure**	**P-value**
**Single-dose**	27.73 ± 13.62	27.31 ± 11.52	0.90*	1289.44 ± 1433.29 (1934♦)	8110.21 ± 10060.76 (7035♦)	0.02*
**Double-dose**	27.96 ± 9.55	27.72 ± 7.76	0.94*	3203.90 ± 2477.08 (3559♦)	3262.18 ± 3059.51 (1769♦)	0.70*
**Surgery **	38.85 ± 20.70	40	NA	12008.99 ± 15752.42 (14689♦)	1681	NA
**Expectant management**	26.93 ± 10.41	31.18 ± 15.28	0.27*	2647.33 ± 5258.32 (1991♦)	2260.15 ± 4747.89 (1358♦)	0.82*
Data shown as Mean ± SD. EP: Ectopic pregnancy, β-hCG: Beta-human chorionic gonadotropin, NA: Not applicable, mm: Millimeter, *Independent sample *t* test,♦IQR: Interquartile range. Among the surgery group, the treatment failed for only one case who had laparoscopy-salpingostomy						

## 4. Discussion 

The current study was performed to compare different EP treatments including surgery, medical treatment and expectant management. EP treatment depends on the hemodynamic stability of patients, β-hCG levels, pregnancy planning, the size of the EP and the presence of fetal cardiac activity (12). Specialists have agreed that medical therapy is not recommended if the initial serum β-hCG values are < 1500 mIU/ml in women with unruptured tubal EP who may be treated by expectant management (13). MTX is a folic-acid antagonist that is accepted as a non-invasive effective treatment for cases with particular criteria (e.g., hemodynamic stability, fertility need) (14).

The SD-MTX method is the most commonly used protocol for cases with EP. The success rate of this protocol has been reported in previous studies to range between 52% and 94% (12, 15). In our study, the success of SD-MTX was 80.46%. Although there are no specific criteria used to recommend SD-MTX, the treatment success may be affected by β-hCG levels, positive fetal cardiac cavity, presence of a yolk sac, and the size of EP (16). It has been observed that the treatment success of the SD protocol decreases when the initial serum β-hCG values are > 2000 mIU/ml (17). Our results are in accordance with those reported in the literature. We demonstrated a significant difference in initial serum β-hCG values between the women who had a successful vs. failed SD treatment (1289.44 ± 1433.29 vs. 8110.21 ± 10060.76 (IQR = 7035) with p = 0.02). In this study, we did not find any significant difference in treatment success between SD and DD medical therapy which is in agreement with the findings reported in previous research. Therefore, we suggest that the SD-MTX protocol should be preferred since the use of the DD-MTX protocol may amplify the side effects, lengthen the hospital stay, cause more anxiety in patients, and also increase medical costs (18).

Surgery is approved as the first option for women who present with signs of tubal rupture and probable intra-abdominal bleeding (19). We found that the women who were selected for surgery had significantly larger ectopic masses and shorter hospital stays. According to the results of this study, we recommend surgery for cases with an ectopic mass size > 34.5 mm or a β-hCG level > 6419 mIU/ml.

Our findings are compatible with previous studies which reported that a high failure rate of medical therapy occurred if the size of the EP mass was > 35 mm (20, 21). Previously it was reported that a serum β-hCG level < 5000 mIU/ml could be considered for successful medical treatment (1, 22). In the current study, the predictive cut-off value for successful DD-MTX was ≤ 6419 mIU/ml, which was higher than prior studies.

In this study, among the management groups, surgery had a 99.35% success rate and so was the most effective method, and DD-MTX, with a 72.09% success rate, was the least. Previous studies have found a success rate from 72-87% for SD- and DD-MTX treatment (23, 24). Some studies have reported that the success rates of DD and multiple dose MTX regimens are higher than that of SD (24); and Song et al., in a prospective study, showed that the SD-MTX method, with the option of second dose administration for the cases with treatment failure, could be an appropriate medical treatment of EP for eligible women (23). In our study, women who were given SD-MTX and DD-MTX treatment did not significantly differ in their size of ectopic mass, β-hCG level or the treatment success; furthermore, the hospital stays in the SD-MTX group were significantly shorter than in the DD-MTX group. We conclude that SD-MTX is preferable to DD-MTX for eligible cases.

The limitation of this study was that characteristics like former EP in the same location, child-bearing characteristics and others that may influence the specialist's opinion on treatment method were not examined. It is recommended to categorize the patients according to different criteria in further studies.

## 5. Conclusion 

Based on our results, surgery is suggested as the best option for cases with a size of ectopic mass > 34.50 mm in diameter or a β-hCG level > 6419 mIU/ml. SD-MTX had a higher success rate and shorter duration of hospitalization compared to the DD-MTX protocol, and so was found to be an efficient and safe alternative. However, further randomized clinical trials with larger sample sizes are required to confirm the current results.

##  Conflict of Interest

The authors declare that there is no conflict of interest.
